# A Case of Myxedema1Read at the Buffalo Academy of Medicine, Section on Pathology, June 21, 1898.

**Published:** 1898-10

**Authors:** J. C. Clemesha

**Affiliations:** M. D., M. R. C. S., L. R. C. P., London; Assistant surgeon Buffalo Eye and Ear Infirmary; 329 Franklin Street


### A CASE OF MYXEDEMA.[1]

BY J. C. CLEMESHA, M. D., M. R. C. S., L. R. C. P., London.
Assistant surgeon Buffalo Eye and Ear Infirmary.

THIS peculiar disease was first described by Sir William Gull as "a cretinoid state supervening in adult life in women," but it was not generally recognised or fully described before the year 1878, when Dr. Ord brought his cases before the clinical society of London.

The prominent features of myxedema are a peculiar swelling of the skin and subcutaneous tissue with dryness and roughness of the

1. Read at the Buffalo Academy of Medicine, Section on Pathology, June 21, 1898.

surface. Pronounced mental deterioration, indicated by dulness, apathy, hebetude, slowness of speech or action. Atrophy or other destructive change of the thyroid gland. Women are far more frequently affected than men and in the majority of cases the disease begins between the ages of thirty and fifty. The chief symptoms of this disease are well brought out in the following case :

Mary M., aged 24 and unmarried, who went to the Buffalo Eye and Ear Infirmary in June, 1898, for "swollen eyelids and tears running over the face." It was in the spring of 1897 when she first noticed a change in her appearance, but it was not until the fall of the same year that she consulted an oculist about her swollen eyelids. Treatment proved of little avail and the swelling and puffiness became more pronounced, so that when seen at the infirmary her face was broadened and her eyelids showed as semi-translucent folds. Her past history was negative, excepting a mild attack of typhoid fever in 1893. Her father died some years ago from typhoid fever, her mother in childbirth. She is an only child.

*Present Condition.*—Her face is broader than in a photograph taken two years ago, the eyelids, upper and lower, are somewhat thickened and show as semi-translucent folds. The alæ nasi are wider and there is a slight flush over each cheek with a peculiar papular eruption. The forehead is swollen, but there is no pitting on pressure. Her general expression is one of heaviness and dulness, her face swollen and expressionless. The eyelids, owing to the puffiness, have a tendency to an oblique direction, as seen in Chinese races, and her hair has become two shades darker, but is neither scanty nor dry, as is usual in cases of myxedema. The tongue and mucous membrane of the mouth show no change, though her voice has altered, having become monotonous, deliberate and slow. Her teeth have decayed rapidly during the past year, which has made their extraction necessary. Her hands have become broader opposite the metacarpal bones, especially the left hand, and rings that she could wear a year ago are now too small; they are always cold and the nails stunted and brittle. The feet are also swollen and the shoes she now wears are half a size larger than those worn a year ago. She takes cold easily and her mental condition has changed. She feels stupid and drowsy and takes a much longer time in which to do her work than formerly.

Temperature, 98.6°; bowels regular; catamenia regular, though more profuse. The urine contains no albumin or sugar; specific gravity, 10.23. The examination of the neck reveals an atrophic condition of the thyroid gland, more pronounced on the right side, where no trace of the glandular tissue can be made out.

The following is taken from a lecture of Victor Horsley, reported in the *British Medical Journal*, Vol. II., 1896 :

Nearly 100 years ago, King, of Guy's hospital, showed by direct experiments that the colloid material of the acini of the gland passed into the lymphatics, and thus, for the first time, was suggested in a tangible form the modern view of the so-called internal secretion of the thyroid gland.

During the subsequent fifty years, and at a time when, with the exception of snake poison, no animal fluid was known to have a very powerful influence on the body when administered in extremely small doses, it is not surprising that the idea that the thyroid gland had any importance at all in the economy was scouted, and that very absurd opinions were formulated to explain its existence and presence in the neck.

The first great step from this step of disregard was the discovery by Schiff, in 1859, that the organ was important to human life. Unfortunately his experiments attracted no attention, and it was not until 1884 that the question was reopened by Schiff and placed upon an accurate basis. At the same time Reverdin and Kocher had established the fact that total removal of the goitre was liable to be followed by certain symptoms, which from the description were recognised to be analogous to myxedema. Attention was drawn to this matter and Horsley undertook an experimental investigation into the question, using monkeys for the purpose. He established beyond question the fact that typical symptoms of myxedema could be produced in a monkey by simple removal of the thyroid gland, and the synchronous examination of the other structures of the neck, for example the nerves, proved that no injury was done to them and that therefore the symptoms were solely and wholly due to the loss of the gland itself. These experiments negatived the theory that myxedema was due to changes in the sympathetic nerve and the influence of the vaso-motor system, and the final destruction of this theory was given by the brilliant discovery of Dr. Murray that this terrible and fatal disease can be cured by the internal administration of that structure, the loss of which had caused the disease.

It would take too long a time to go into the special points in the functional activity of the thyroid gland, the microscopical changes, but we may state briefly that in myxedema the thyroid gland atrophies and there is found a round-celled infiltration of the remnants of the lobes and acini, with changes in the epithelial cells. Finally, nothing but dense fibrous tissue is left.

In regard to the treatment of myxedema Mr. Horsley had suggested that this disease might be cured by transplantation of the thyroid gland, but thyroid extract was first suggested by Dr. Murray in 1891, and given to a woman who had suffered from myxedema for five years. Four years after the treatment was commenced she was free from myxedema and had long been so.

The patient whose case has been here described has now been taking grains xxv. thyroid extract per day for the last three months. Her appearance has undergone a marked change for the better. The puffiness is gone from the forehead and eyelids, the appearance of breadth from the features. Mentally she is much brighter and declares she feels a different woman.

Before closing it might be well to consider some of the effects of the administration of thyroid extract. There is an increase in tissue, metabolism and often, at first, a large increase in the urine passed and a large increase in the urea excreted; hence of late thyroid extract has been largely used in the treatment of obesity.

Increased rapidity of the heart's action is perhaps the most constant of the effects observed from thyroid administration and the effects of the remedy can hardly be said to be manifested unless the pulse rises by several beats per minute. Sometimes palpitation and irregularity are observed, hence it is necessary in the administration of the drug to begin with a small dose and gradually increase it. Among the symptoms produced by too large doses are headache, pains in the limbs, nausea, diarrhea and palpitation.

329 FRANKLIN STREET.

